# An overview on COVID-19: reality and expectation

**DOI:** 10.1186/s42269-020-00341-9

**Published:** 2020-06-01

**Authors:** Manal A. Hamed

**Affiliations:** Department of Therapeutic Chemistry, National Research Centre, El-Buhouth St., Dokki, Giza, Egypt

**Keywords:** SARS-COV-2, COVID-19, Drugs, Vaccines, Plasma transfusion, Herbal treatment

## Abstract

Recently, severe acute respiratory syndrome coronavirus 2 (SARS-COV-2), commonly known as coronavirus disease-2019 (COVID-19) has rapidly spread across China and around the world. By the declaration of WHO, COVID-19 outbreak considered as a public health problem of international concern. The aim of this study is to provide a comprehensive view on COVID-19 and the future expectations to control virus progression. Patients with liver disease, diabetes, high blood pressure, and obesity are more susceptible to the incidence of COVID-19 infection. So, there is a rapid need for disease diagnosis, vaccine development, and drug discovery to detect, prevent, and treat this sudden and lethal virus. Real-time polymerase chain reaction (RT-PCR) is considered as a rapid, accurate, and specific tool for disease diagnosis. Under this emergency situation that the world facing against COVID-19, there are about 15 potential vaccine candidates tested globally based on messenger RNA, DNA-based, nanoparticle, synthetic, and modified virus-like particle. Certain drugs that are clinically approved for other diseases were tested against COVID-19 as chloroquine, hydroxychloroquine, ivermectin, favipiravir, ribavirin, and remdesivir. Convalescent plasma transfusion and traditional herbal medicine were also taken into consideration. Due to the absence of effective treatment or vaccines against COVID-19 so far, the precautionary measures according to WHO’s strategic objectives are the only way to confront this crisis. Governments should adopt national medical care programs to reduce the risk of exposure to any future viral outbreaks especially to patients with pre-existing medical conditions.

## Introduction

Coronaviruses have been recognized for over 50 years. The word “corona” has many different meanings, but it was the sun that the virologists had in mind when they chose the name coronaviruses by comparing the characteristic projections on the outside of the virus with the solar corona. Coronaviruses cause severe acute respiratory syndrome (SARA) leading to death in most cases. Coronaviruses are single-stranded RNA viruses, about 120 nm in diameter. They are susceptible to mutation and are therefore highly diverse. They mainly infect human and non-human mammals and birds. The first coronaviruses found to infect humans were called 229E and OC43 in 1968, but they caused very mild infections until the outbreaks of severe acute respiratory syndrome (SARS-COV-1) in 2003, MERS (Middle Eastern respiratory syndrome) in 2012 and SARS-COV-2 (COVID-19) in 2019 which caused serious human infections (Aronson 2020).

**Fig. 1:**
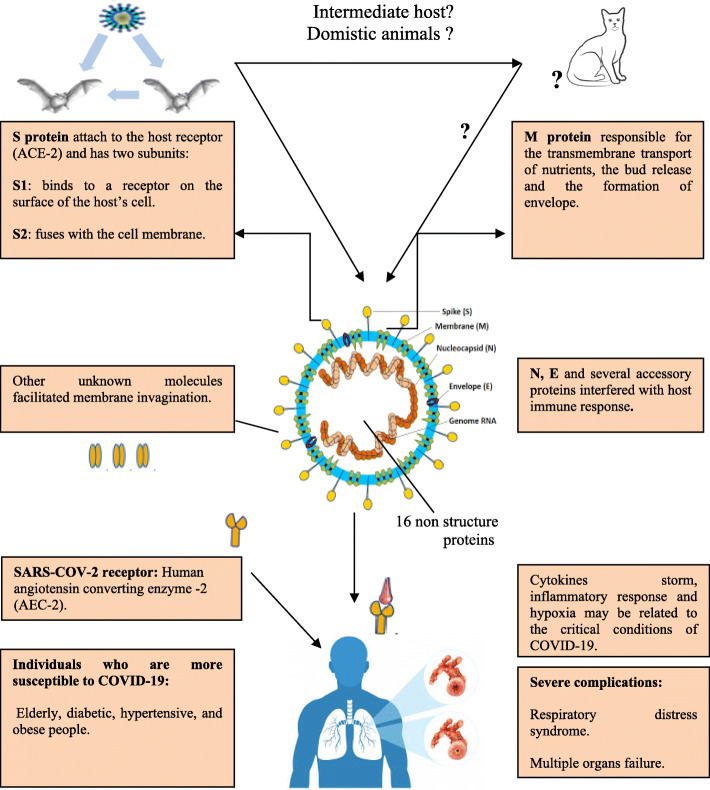
Origin, transmission, and infection of COVID-19

The virus that causes COVID-19 (SARS-COV-2) is thought to have originated in bats and then spread to humans through contamination of meat sold in China’s meat markets with wild animals’ wastes. The coronavirus syndrome is caused by spike glycoproteins, which are necessary for the viruses to enter host cells. The spike has two subunits: one subunit, S1, binds to a receptor on the surface of the host’s cell; the other subunit, S2, fuses with the cell membrane. The cell membrane receptor is a form of angiotensin converting enzyme (ACE-2). Briefly, the S1 subunit of the spike binds to the ACE-2 enzyme on the cell membrane surface, the host transmembrane serine protease (TMPRSS2) activates the spike and cleaves ACE-2, and the TMPRSS2 acts on the S2 subunit, facilitating fusion of the virus to the cell membrane and then enters the cell. Inside the cell, the virus is released from endosomes by acidification or the action of an intracellular cysteine protease (cathepsin), where it replicate and affect many organs especially the lung (Fig. 1) (Zhong et al. 2003; Zaki et al. 2012; Aronson 2020; ECDC 2020a, 2020b; Chen et al. 2020; Huang et al. 2020; Zhu et al. 2020).

The aim of this study is to provide an overview on a newly coronavirus appeared in 2019 with future expectations to counteract its severity and progression.

### Incidence, prevalence, and epidemic situation of COVID-19

On 31 December 2019, a cluster of pneumonia cases of unknown etiology was reported in Wuhan, Hubei Province, China. On 9 January 2020, China reported a novel coronavirus as the causative agent of this outbreak (coronavirus disease-2019) which is commonly known as COVID-19 (ECDC 2020a). The initial reported cases in Wuhan mentioned that the majority of coronavirus cases were males with a median age of 55 years and linked to the Huanan Seafood Wholesale Market (Chen et al. 2020). Most of these cases had similar symptoms like fever, cough, fatigue, myalgia, and diarrhea. Many cases developed pneumonia, and some had severe and fatal respiratory diseases (Huang et al., 2020, b).

On 25 March 2020, more than 416,916 cases of COVID-19 were reported worldwide by more than 150 countries, where the latest country confirmed a COVID-19 case outside of China is Egypt (ECDC 2020a). On 8 April 2020, 1,391,890 cases of COVID-19 have been reported, including 81,478 deaths (ECDC 2020a).

The novel COVID-19 coronavirus is a zoonotic β-coronavirus that belongs to the subgenus: sarbecovirus of the Orthocoronavirinae subfamily (Zhu et al. 2020). The severe acute respiratory syndrome coronavirus (SARS-COV-1) and Middle East respiratory syndrome coronavirus (MERS-COV) are also zoonotic β-coronaviruses associated with the outbreaks in 2003 and 2012, respectively (Zhong et al., 2003; zaki et al. 2012). Although the pathogenicity of COVID-19, SARS-COV-1, and MERS-COV are 3%, 10%, and 40%, respectively (Chen 2020), COVID-19 has 1.4–5.5 high transmission rate than SARS-COV-1 (2–5) and MERS-COV (< 1) (Chen 2020). The mean incubation period for COVID-19 is widely varies between individuals (5.2–14 days), so further investigations are needed to better understand the viral shedding time to inform optimal specimen collection for diagnosis (Li et al. 2020).

### COVID-19 patients with pre-existing medical conditions

It was recorded that 2–10% of patients with COVID-19 had SARS-COV-2 RNA in stool and blood samples which implicated the possibility of viral exposure in the liver (Zhang et al., 2020). This was supported by the same authors through a marked elevation of liver function enzymes (aspartate and alanine aminotransferases) in COVID-19 patients. In addition, gamma glutamyl transferase (GGT), a diagnostic biomarker for cholangiocyte injury, was elevated in 30 (54%) of 56 patients with COVID-19. Due to the ubiquitous distribution of the main viral entry receptor, namely, angiotensin converting enzyme 2 (ACE2), SARS-COV-2 causes a systemic disease, with possible involvement of the heart, the liver, the pancreas, and the kidneys, as well as determines alterations in circulating lymphocytes and the immune system (Huang et al., 2020, b). Chai et al. (2020) stated that ACE2 expression was enriched in cholangiocytes patients. This observation is indicating that SARS-COV-2 might directly bind to ACE2-positive cholangiocytes to dysregulate liver function. Moreover, Zhang et al. (2020) postulated that patients with liver cirrhosis or liver cancer might be more susceptible to SARS-COV-2 infection because of their systemic immunocompromised status.

Zhao et al. (2020) reported a case of COVID-19 with a history of co-infection of HIV-1 and HCV that showed delayed antibody response. This case highlights the possible influence of HIV-1-induced immune dysfunction on the immune responses to and clearance of SARS-COV-2. One potential explanation is that he was taking anti-HIV-1 agents which had been reported to have anti-SARS-COV-2 effects (Martinez 2020). Another possibility is that the activated type I interferon (IFN-I) may help suppress SARS-COV-2. Previous study has shown that HIV-1 infection may induce high levels of IFN-I, which may to some extent clear SARS-COV-2 infection, thus leading to persistently undetectable RNA (Tang et al. 2020b).

Diabetes and uncontrolled glycaemia were reported as significant predictors of severity and deaths in patients infected with different viruses including the pandemic influenza A (H1N1), SARS-COV, and MERS-COV (Yang et al. 2006; Banik et al. 2016). In the current SARS-COV-2 pandemic, Wu and McGoogan (2020) showed that patients with chronic diseases, including diabetes, were at higher risk for severe COVID-19 infection and mortality.

Infection of SARS-COV-2 in those with diabetes possibly triggers higher stress conditions, with greater release of hyperglycemic hormones as glucocorticoids and catecholamines, leading to increased blood glucose levels and abnormal glucose variability (Wang et al. 2020a). In another study, patients with type 2 diabetes and COVID-19 were reported that they suffered from hypoglycemia (Zhou and Tan 2020). Hypoglycemia has been shown to mobilize pro-inflammatory monocytes, increase platelet reactivity, and therefore contributing to a higher cardiovascular mortality (Iqbal et al. 2019). Yet, it remains unknown how the inflammatory and immune response occurs in these patients, as well as whether hyper or hypoglycemia may alter the SARS-COV-2 virulence, or the virus itself interferes with insulin secretion or glycemic control. Wang et al. (2020b) explained that the metabolic disorders in diabetic patients reduce immune response by impairing macrophage and lymphocyte functions which might subsequently render individuals more susceptible to infectious viral disease complications.

Hypertension is a major risk factor of mortality worldwide, and its importance is further emphasized in the context of COVID-19. Patients with severe COVID-19 infections commonly are older and have a history of hypertension. Almost 75% of patients who have died in the pandemic in Italy and China had hypertension (Kreutz et al. 2020). As previously mentioned that ACE-2 level increased in SARS-COV-2 patients to facilitate viral entry to host cells, Wan et al. (2020) added that the expression of ACE2 is also increased in patients with hypertension and diabetes due to treatment with ACE-2 increasing drug that are at high risk of COVID-19 and therefore should be treated with ACE inhibitors or angiotensin II type-I receptor blockers (ARBs). Leggio et al. (2017) and Oyekale (2019) added that there is a significant association between obesity and hypertension.

Until now, there is no clear evident suggesting direct association between obesity and surveillance of COVID-19. Indirect association between obesity and COVID-19 is monitored, where obesity is associated with decreased expiratory reserve volume, functional capacity, and respiratory system compliance. In patients with increased abdominal obesity, pulmonary function is further compromised by decreased diaphragmatic excursion, thus making ventilation more difficult. So, Dietz et al. (2020) mentioned the impact of obesity on pulmonary function that increases the risk of COVID-19 in obese patients. Furthermore, increased inflammatory cytokines associated with obesity may contribute to the increased morbidity associated with obesity in COVID-19 infections (Dietz et al. 2020). Therefore, patients with obesity were more likely to need an intensive care unit for acute lung injury and might have prolonged mechanical ventilation and hospital stay, compared with patients with normal weight. In addition to the detrimental effects on lung function, obesity is a confirmed cause of diabetes and cardiovascular disease and higher overall mortality (Qingxian et al. 2020).

### Clinical diagnosis

By the declaration of the World Health Organization that COVID-19 outbreak is a public health emergency of international concern, there is a rapid need of disease diagnosis, vaccine development, and drug discovery to detect, prevent, and treat COVID-19. Thereafter, real-time PCR (RT-PCR) is considered as a golden tool for diagnosing (Chu et al. 2020; Corman et al. 2020). The period and type of specimen collected for RT-PCR play an important role in the diagnosis of COVID-19. It was found that the respiratory specimens were positive for the virus, while serum was negative in the early period. It has also suggested that patients have high levels of virus in the early days of viral infection rather than in the late stage (Pang 2020). Rapid collection and testing of specimens are important and should be guided by a laboratory expert (Li et al. 2020). There are seven rapid RT-PCR diagnostic kits available on the market for COVID-19. Six of these kits are for research purposes, while only one from Beijing Genome Institute (BGI) (China) is approved for clinical diagnosis (Pang et al. 2020). Additionally, there are two kits (BGI, China and Veredus, Singapore) that have the capability to detect COVID-19 and another pathogens using sequencing and microarray technologies, respectively (Pang et al. 2020).

### Vaccine development and immune response

With the emergency situation that the world facing against COVID-19, there are about 15 potential vaccine candidates tested globally based on messenger RNA, DNA-based, nanoparticle, synthetic, and modified virus-like particle. Coronavirus expresses several structural proteins, including nucleocapsid, membrane, envelope, and spike (S) proteins (Peiris et al. 2004). Each one of them may serve as antigen to induce neutralizing antibodies and protective responses. Prior to identification of the protein that contains the major neutralizing epitopes, the inactivated virus may be used as a vaccine because it is easy to generate whole killed virus particles. Once the neutralizing epitopes are identified, the inactivated virus vaccine should be replaced by vaccines based on fragments containing neutralizing epitopes as they become more effective. The virus was inactivated by formaldehyde, UV light, and β-propiolactone to induce virus-neutralizing antibodies in animals (He et al. 2004; Qu et al. 2005). The first inactivated vaccine is being tested in the clinical trials in China. The safety of the inactivated vaccine production is limited due to the highly risk of infection exposure to workers during handling of concentrated live COVID-19. Additionally, the incomplete virus inactivation may cause virus outbreaks of the vaccinated people. Moreover, some viral proteins may cause harmful immune or inflammatory responses and causing coronavirus-like diseases (Wang and Lu 2004).

The S protein of the virus, a type I transmembrane glycoprotein, is responsible for virus binding, fusion, and entry and is a major inducer of neutralizing antibodies (Holmes 2003). S protein consists of a signal peptide and 3 domains (extracellular, transmembrane, and an intracellular domain). Its extracellular domain consists of 2 subunits: S1 and S2 (Holmes 2003). The S1 subunit is responsible for virus binding to the receptor, angiotensin-converting enzyme 2 (Li et al. 2003). A fragment located in the middle region of the S1 subunit is the receptor-binding domain for angiotensin-converting enzyme 2 (Wong et al. 2003). The S2 subunit, which contains a putative fusion peptide and 2 heptad repeats, is responsible for fusion between the virus and the target cell membranes. Infection by coronavirus is initiated by binding of the receptor binding domain in the viral S protein S1 subunit to angiotensin-converting enzyme 2 on target cells. This forms a fusogenic core between the 2 heptad repeats regions in the S2 domain that brings the viral and target cell membranes into close which results in virus fusion and entry (Liu et al. 2004; Tripet et al. 2004). This remark indicates that the S protein may be used as a vaccine to induce antibodies for blocking virus binding and fusion.

### COVID-19 and genetic heterogeneity

In case of COVID-19, Tang et al. (2020a) postulated that one non-synonymous SNP (single nucleotide polymorphism) was detected in gene encodes S (spike) protein in COVID-19. This SNP replaced serine amino acid by leucine, and for this reason, they called the two new strains: (S strain) and (L Strain). The former (S) is the wild type which is milder while the latter (L) is the novel one which resulted in high binding affinity between SARS-COV-2 virus with angiotensin-converting enzyme 2 receptor in human cells. The L strain is responsible for pandemic infection and never be seen in any previous version of corona (SARS, MERS) and also in bat, pangolin, civet, camel.

Many studies have recently confirmed the genetic similarity (96.2%) between COVID-19 (SARS-COV-2) and a bat SARS-related coronavirus (SARS-COV-1). It has also been confirmed that the SARS-COV-2 uses the same receptor: the angiotensin converting enzyme 2, as the SARS-COV-1 (Gralinski et al. 2020; Paraskevis et al. 2020).

Bacillus Calmette-Guérin (BCG) is a well-known vaccine against tuberculosis (TB). Hegarty et al. (2020) found that the BCG vaccinated healthy controls re-challenged with yellow fever virus showed improvement in anti-viral immunity and decrease in viral loads. Accidentally, the same authors found that the incidence of COVID-19 in 131 countries applied the national programs of BCG vaccination was 38.4 per million compared to 358.4 per million in countries not applied such a vaccination program. Also, the death rate was 4.28/million in countries with BCG programs compared to 40/million in countries without such a program. However, there is no clear evidence that the BCG vaccine protects people against infection with COVID-19 virus.

### Therapeutic potential of certain drugs

**Fig. 2:**
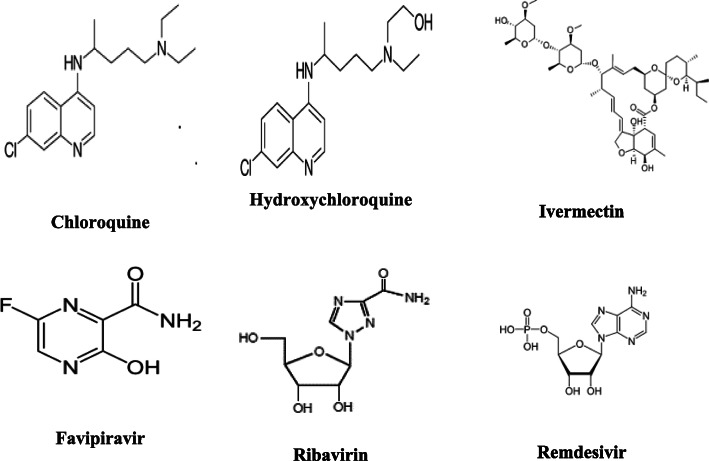
Tested drugs for COVID-19 treatment

Based on the current lack of an approved and effective vaccine for COVID-19, it is important to evaluate certain drugs (Fig. 2) that are clinically approved for other diseases against the new coronavirus. Chloroquine (CQ) and its derivative, hydroxychloroquine (HCQ), are considered as prophylactic drugs against malaria and as treatments for autoimmune diseases (Hu et al. 2020). Previous studies have revealed that chloroquine has therapeutic activity against viruses, including human coronavirus in animal models and SARS-COV-1 in cell culture studies (Savarino et al. 2003; Keyaerts et al. 2009). The mechanisms through which chloroquine may act to attenuate SARS-COV-2 infections may be valuable for identifying new prophylactic and therapeutic agents. Chloroquine is a weak base that becomes entrapped in membrane-enclosed low pH organelles, and interfering with their acidification (Hempelmann 2007). In case of viral infection, chloroquine may induced antiviral effects by inhibition of pH-dependent viral fusion/replication and prevention of viral envelope glycoprotein as well as host receptor protein glycosylation. Chloroquine may also inhibit virion assembly in endoplasmic reticulum-Golgi intermediate compartment-like structures. Additionally, chloroquine may directly affect the host by attenuating the expression of pro-inflammatory factors and receptors that can induce acute respiratory syndrome, which is primarily responsible for coronavirus-associated mortality (Hu et al. 2020). Guastalegname and Vallone (2020) concluded that no acute virus infection has been successfully treated by CQ/HCQ in human. Chloroquine and hydroxychloroquine did not show any anti SARS-COV-2 effect on in vivo. As the pathogenesis of COVID-19 is still unknown, therefore, the immune effect provoked by chloroquine or hydroxychloroquine administration in COVID-19 patients is unpredictable.

Ivermectin is an FDA-approved broad spectrum anti-parasitic agent. In recent years, it shows anti-viral activity against a broad range of viruses in vitro. Studies on SARS-COV proteins have revealed a potential role for importin-α/β1 (IMPα/β1) that recognize nuclear localization sequences during infection in signal-dependent nucleocytoplasmic shutting of the SARS-COV nucleocapsid protein (Wulan et al. 2015), and that may impact host cell division (Wurm et al. 2001). In addition, the SARS-COV accessory protein (ORF6) has been shown to antagonize the antiviral activity of the signal transducer and activator of transcription (STAT) factor by sequestering IMPα/β1 on the rough ER/Golgi membrane (Frieman et al. 2007). Therefore, the ivermectin nuclear transport inhibitory effect may be effective against SARS-COV-2. Caly et al. (2020) added that ivermectin acted as an inhibitor of SARS-COV-2, with a single addition to Vero-hSLAM cells 2 h post infection and showed ~ 5000-fold reduction in viral RNA at 48 h.

Favipiravir (T-705; 6-fluoro-3-hydroxy-2-pyrazinecarboxamide) was discovered by chemical modification of a pyrazine analog initially screened by in vitro anti-influenza virus activity in cells. Favipiravir is an anti-viral agent that selectively and potently inhibits the RNA-dependent RNA polymerase (RdRp) of RNA viruses. Favipiravir undergoes an intracellular phosphoribosylation to be an active form, favipiravir-RTP (favipiravir ribofuranosyl-5B-triphosphate), which is recognized as a substrate by RdRp, and inhibits the RNA polymerase activity. The mechanism of action of favipiravir depends on inhibition of viral replication genome, which manifested in the middle of viral proliferation cycle. The anti-viral activity of favipiravir was attenuated in the presence of purine bases, indicating its competition with purine rather than pyrimidine nucleosides. Therefore, favipiravir with these unique profiles will be a promising therapeutic agents of RNA viruses (Furuta et al. 2017). Favipiravir was approved in Japan as an influenza antiviral drug in 2014. The drug is considered to use when there is an outbreak of influenza virus infections in which other influenza drugs are either not effective or insufficiently effective. The drug may potentially have an antiviral effect on the novel coronavirus as it is classified into the same type of a single-stranded RNA virus as the common influenza virus. However, the clinical application of favipiravir to treat coronavirus disease (COVID-19) is under examination for clear evidence of its efficacy and safety (FUJIFILM 2020).

Nitazoxanide is originally developed as an antiprotozoal agent treatment of intestinal infections caused by *Cryptosporidium parvum*. Nitazoxanide is a broad-spectrum antiviral agent undergoing clinical development for treatment of influenza and other viral respiratory infections (Rossignol 2016). It is presently undergoing phase 3 clinical development for treating acute uncomplicated influenza (Rossignol 2014). It exhibits in vitro activity against MERS-COV and other coronaviruses by inhibiting expression of the viral protein. Nitazoxanide also suppresses production of pro-inflammatory cytokines in peripheral blood mononuclear cells and interleukin 6 production. As it is used extensively in clinical trials, nitazoxanide may be tested as a treatment against SARS-COV-2 (Rossignol 2014).

Momattin et al. (2013) mentioned that there were seven reports of the use of ribavirin as a treatment in SARS patients, while two studies showed improvements of symptoms in 71.4%-80% of patients. Khalili et al. (2020) proved the utility of ribavirin in the treatment of COVID-19 patients. The major problem with ribavirin was the incidence of hemolysis. Interferon treatment led to improvements in clinical symptoms in SARS patients, however, no advantage of ribavirin over interferon in patients with SARS. The addition of lopinavir/ritonavir to ribavirin regimen was associated with improved clinical outcome and reduces the death rate comparing to ribavirin regimen alone (Momattin et al. 2013).

Remdesivir has been recently recognized as a promising antiviral drug against a wide array of RNA viruses (including SARS/MERS-COV2) infection in cultured cells, mice, and non-human primate models (Wang et al. 2020c). Remdesivir is an adenosine analogue, which incorporates into nascent viral RNA chains and results in pre-mature termination (Warren et al. 2016). Wang et al. (2020c) showed that remdesivir functioned at a stage post virus entry, which is in agreement with its putative antiviral mechanism as a nucleotide analogue. The same authors showed that the EC90 value of remdesivir against COVID-19 in Vero E6 cells was 1.76 μM, suggesting its working concentration is likely to be achieved as in non-human primates studied before. Additionally, remdesivir also inhibited virus infection efficiently in a human liver cancer Huh-7 cells, which is sensitive to COVID-19.

### A possible new concept of therapy

As previously mentioned, the novel coronavirus is a positive-stranded RNA with structural proteins as spike protein (S), envelope protein (E), membrane protein (M), nucleocapsid phosphoprotein, and transcribed non-structural proteins as orf1ab, ORF3a, ORF6, ORF7a, ORF10, and ORF8 (Liu and Li 2020). The same authors showed that the ORF8 and surface glycoprotein could bind to the porphyrin, respectively. At the same time, orf1ab, ORF10, and ORF3a proteins attack the heme on the 1-beta chain of hemoglobin to dissociate the iron to form the porphyrin. This attack causes less hemoglobin that carry oxygen and carbon dioxide. Therefore, the lung cells have intense poisoning and inflammation due to its inability to exchange carbon dioxide and oxygen easily. According to these observations, chloroquine could prevent orf1ab, ORF3a, and ORF10 to attack the heme and inhibit the binding of both ORF8 and surface glycoproteins to porphyrins that effectively relieve the symptoms of respiratory infection. Favipiravir could inhibit the envelope protein and ORF7a protein bind to porphyrin, prevents the virus from entering host cells, and catching free porphyrins. As Liu and Li (2020) confirmed this hypothesis that the novel coronavirus is dependent on porphyrins, it may lead scientists to discover new drugs or confirmed chloroquine and favipiravir for disease prevention or clinical treatment depending on inhibition of viral attach to heme to prevent the state of hypoxia.

### Convalescent plasma transfusion therapy

The therapeutic use of convalescent plasma donated by patients recovered from COVID-19 might play a role in the efforts to find a possible treatment for COVID-19 (ECDC 2020b). The use of convalescent plasma was recommended before as an important treatment during outbreaks of Ebola virus, Middle East respiratory syndrome coronavirus, SARS-COV-1, H5N1 avian influenza, and H1N1 influenza (Zhou et al. 2007; Hung et al. 2011; Kraft et al. 2015). In a study involving patients with pandemic influenza (H1N1) and SARS virus, treatment of severe infection with convalescent plasma was associated with reduced respiratory viral load, serum cytokine response, and mortality (Cheng et al. 2005; Hung et al. 2011). Accordingly, these findings raise the hypothesis that use of convalescent plasma transfusion could be beneficial in patients infected with SARS-COV-2. However, the risk of COVID-19 transmission via plasma transfusion must be seriously consider through the uncertainties viraemia during the incubation period, an asymptomatic course of infection, viral plasma contamination, patients with liver or kidney transplantation, and the 14 days of recovery period of donor after symptom resolution with negative results of repeated antiviral tests (Liu and Li et al. 2020). Therefore, precautionary measures are suggested to mitigate these risks.

### Traditional herbal medicine

The close similarity between COVID-19 and SARS-COV-1 through their rapid transmission, their genome sequences, and the common entrance of their spike protein to alveolar epithelial cells through binding with ACE-2 enzyme receptor, all these encourage Chinese researchers to treat COVID-19 patients with traditional Chinese medicine (TCM) used for SARS-COV-1 before (Yang et al. 2020).

As 3-chymotrypsin-like protease (3CLpro) is a vital compound for virus replication, thereafter, water extract of *Houttuynia cordata*, flavonoid extracted from litchi seeds, beta-sitosterol extracted from the root of *Isatis indigotica*, naturally occurring sinigrin, indigo aloe-emodin, hesperetin, quercetin, epigallocatechin gallate, gallocatechin gallate, herbacetin, rhoifolin, and pectolinarin were able to inhibit the SARS 3CLpro activity. Moreover, flavonoids compounds as herbacetin, isobavaschalcone, quercetin 3β-D-glucoside, and helichrysetin had the potential to block the 3CLpro activity of MERS-COV (Lin et al. 2005; Lau et al. 2008; Gong et al. 2008; Luo et al. 2009; Fung et al. 2011; Nguyen et al. 2012; Jo et al. 2019 and 2020).

In addition, many studies focused on the efficiency of TCM to target angiotensin converting enzyme 2 to prevent the infection of COVID-19 like emodin from genus *Rheum* and *Polygonum*, baicalin from in *Scutellaria baicalensis*, nicotianamine from soybean, tetra-O-galloyl-β-D-glucose from *Galla chinensis*, and luteolin from *Veronicalina riifolia* (Yi et al. 2004; Ho et al. 2007; Deng et al. 2012; Takahashi et al. 2015; Wang et al. 2016).

Moreover, the anti-inflammatory herbs as *Lonicerae japonicae* Flos, *Scutellariae radix*, *Fructus Forsythiae*, and *Curcuma longa* as well as the biologically active compounds hesperidin and naringenin isolated from citrus fruits could reduce the severity and mortality rate by their effect on the transcriptional and translational levels of inflammatory cytokines TNF-α, IL-1β, and IL-6 (Chen et al. 2002; Gao et al. 2014; El-Rafie et al. 2014; Hamed et al. 2019 and 2020; Lu 2020; Zou 2020).

### Challenges for control of the epidemic

Due to the absence of effective treatment or vaccines against COVID-19, the precautionary measures are the only way to confront this crisis. Transmission of coronaviruses from contaminated dry surfaces has been postulated including self-inoculation of mucous membranes of the nose, eyes, or mouth (Kampf et al. 2020). Human coronaviruses can remain infectious on inanimate surfaces for up to 9 days. So, various types of biocidal agents such as hydrogen peroxide, alcohols, sodium hypochlorite, or benzalkonium chloride are used worldwide for disinfection mainly in healthcare settings (Kampf 2018). Social isolation, distancing, or quarantine of entire communities may be useful. Nonetheless, these measures should be implemented in a prudent fashion while considering their cost efficiency. Also, there is a real need to avoid the epidemic wave that would saturate the capacity of health services. Moreover, it is important to note that collective infection control measures can actually reduce the frequency of infection (Raoult et al. 2020).

### WHO intervention for epidemic control

The 78th situation report of WHO postulated the strategic objective control for COVID-19 which are (1) interrupt human-to-human transmission including reducing secondary infections among close contacts and health care workers, preventing transmission amplification events, and preventing further international spread; (2) identify, isolate, and care for patients early, including providing optimized care for infected patients; (3) identify and reduce transmission from the animal source; (4) address crucial unknowns regarding clinical severity, extent of transmission and infection, treatment options, and accelerate the development of diagnostics, therapeutics and vaccines; (5) communicate critical risk and event information to all communities and counter misinformation; and (6) minimize social and economic impact through multisectoral partnerships (WHO 2020).

This can be achieved through a combination of public health measures, such as rapid identification, diagnosis and management of the cases, identification and follow-up of the contacts, infection prevention and control in health care settings, implementation of health measures for travelers, awareness-raising in the population, and risk communication (WHO 2020).

## Conclusions

COVID-19 outbreak is a public health emergency of international concern. Patients with liver disease, diabetes, high blood pressure, and obesity are more susceptible to the incidence of COVID-19 infection. Disease diagnosis, vaccines, and drug discovery are essential to control this pandemic situation. RT-PCR is considered as the most accurate and specific technique for disease detection besides the CT imaging. Many potential vaccine candidates and drugs are tested against COVID-19. Plasma transfusion and herbal medicine were also considered to control this new coronavirus. Due to the absence of effective treatment or vaccines against COVID-19 so far, the precautionary measures according to WHO’s strategic objectives are the only way to confront this crisis. Governments should adopt national medical care programs to reduce the risk of exposure to any future viral outbreaks especially to patients with pre-existing medical conditions.

## Data Availability

Not applicable.
